# A low androgenic state inhibits erectile function by suppressing endothelial glycosides in the penile cavernous tissue of rats

**DOI:** 10.1093/sexmed/qfae039

**Published:** 2024-06-15

**Authors:** Wei Zhou, Jun Jiang, Rui Jiang

**Affiliations:** Department of Urology, The Affiliated Hospital of Southwest Medical University, Luzhou, 646000, China; Department of Urology, Hejiang County Traditional Chinese Medicine Hospital, Luzhou, 646000, China; Department of Thyroid Surgery, The Affiliated Hospital of Southwest Medical University, Luzhou, 646000, China; Department of Urology, The Affiliated Hospital of Southwest Medical University, Luzhou, 646000, China

**Keywords:** androgen, endothelial glycosides, erectile function, penis

## Abstract

**Background:**

The endothelial glycocalyx is an important barrier that protects the structure and function of endothelial cells. Androgen deficiency is a common factor that causes structural and functional impairment of endothelial cells.

**Aim:**

To investigate changes in the endothelial glycocalyx in the penile corpus cavernosum of the rat with low androgen status and its relationship with erection function.

**Methods:**

Eighteen 10-week-old Sprague-Dawley male rats were randomly divided into 3 groups (n = 6 each): sham operation, castration, and castration + testosterone replacement. The maximum intracavernosal pressure/mean arterial pressure of the penis was measured after modeling for 4 weeks. The expression levels of endothelial nitric oxide synthase (eNOS), phospho-eNOS, syndecan 1, heparanase, and nitric oxide in penile cavernous tissue and the serum levels of heparan sulfate, hyaluronic acid, tumor necrosis factor α, and interleukin 6 were determined. Transmission electron microscopy was used to observe the ultrastructure of the endothelial glycocalyx in penile tissue.

**Outcomes:**

The thickness of the endothelial glycocalyx in the penile corpus cavernosum of castrated rats was significantly lower than that of the control group.

**Results:**

In the castrated rats, the endothelial glycocalyx thickness, syndecan 1 level, ratio of phospho-eNOS to eNOS, nitric oxide level, and maximum intracavernosal pressure/mean arterial pressure (3 V, 5 V) were significantly lower than those in the sham group (*P* < .05). The expression of heparanase and the serum levels of tumor necrosis factor α and interleukin 6 were significantly higher in the castrated group than in the sham group (*P* < .05).

**Clinical Translation:**

Upregulating the expression of the endothelial glycocalyx in the penile corpus cavernosum may be a new method for treating erectile dysfunction caused by low androgen levels.

**Strengths and Limitations:**

This study confirms that low androgen status promotes the breakdown of the endothelial glycocalyx. However, further research is needed to determine whether androgens are related to the synthesis of the endothelial glycocalyx.

**Conclusion:**

Low androgen status may suppress the level of nitric oxide in the cavernous tissue of the penis via impairment of the endothelial glycocalyx, resulting in inhibited erection function in rats.

## Introduction

Erectile dysfunction (ED) refers to the inability of the penis to achieve or maintain an erection, which prevents satisfactory sexual intercourse.^1^ More than 150 million men worldwide are affected by ED, and it is projected that the number will exceed 320 million by 2025.^2^ Chronic diseases such as androgen deficiency, aging, diabetes, and cardiovascular disease are common causes of ED.^3^ First-line treatment for ED often involves the use of phosphodiesterase type 5 inhibitors (PDE5Is). For patients with androgen deficiency, the effectiveness of PDE5I treatment is only 35%, and combination therapy with androgen can increase the effectiveness of PDE5I by approximately 10%.^4^^,^^5^ However, treating the erectile function of individuals who need to maintain low androgen levels, such as patients with prostate cancer who are undergoing androgen deprivation therapy, is a challenge worthy of more research attention.

The endothelial glycocalyx is a lumen-like structure in the inner cavity of the vascular wall. This structure consists of core proteins that are the main structural links to a variety of proteoglycans and glycosaminoglycans and attached plasma proteins, forming the endothelial surface layer.^6^ The endothelial surface layer is crucial for maintaining functional vascular barriers, microcirculation, and organ perfusion.^7^ The endothelial glycocalyx is degraded in individuals with diabetic retinopathy and arteriolar hypertension. The breakdown products of hyaluronic acid (HA) and heparin sulfate (HS) are released into the serum and causing inflammatory reactions that lead to endothelial cell dysfunction and increased vascular permeability. Studies have shown that inhibiting endothelial glycocalyx degradation by upregulating intravascular proteins can partially restore vascular function in the brain and retina.^8–12^

Low androgen status is often accompanied by a stenosis of the vascular lumen in the penile cavernous tissue, as well as apoptosis and pyroptosis of endothelial cells.^11^^,^^13^ However, alterations of the endothelial glycocalyx within the rat corpus cavernosum with low androgen status are currently unclear. The goal of our study is to determine if low levels of testosterone affect the endothelial glycosides in the penile corpus cavernosum of rats to inhibit erectile function.

## Methods

### Experimental animal model establishment

Eighteen 9-week-old healthy male Sprague-Dawley rats were acclimatized for 1 week and then randomly divided into 3 experimental groups (n = 6 each): sham, castration, and castration + testosterone replacement. All experimental animals were purchased from the SPF Grade Animal Experiment Center of Southwest Medical University. After intraperitoneal injection of anesthesia (1% pentobarbital sodium, 40 mg/kg), the skin of the sham group was incised along the longitudinal scrotal septum. The skin of the castration group was incised along the longitudinal scrotal septum, and the testes and epididymides were excised. The castration + testosterone group was given subcutaneous injections of testosterone propionate (3 mg/kg; Renjian Pharmaceutical Co, Ltd) every other day after castration.^13^ The rats were fed at a fixed time, and all rats were free to eat and drink water. Blood glucose was examined weekly through tail vein sampling.^14^ All experimental procedures were performed in accordance with the *Guide for the Care and Use of Laboratory Animals* (National Institutes of Health publication 85-23, revised 1996) and were approved by the Ethics Committee for Animal Experiments of Southwest Medical University (SWMU20230009).

### Maximum intracavernosal pressure/mean arterial pressure

After the rats were anesthetized with an intraperitoneal injection of 1% pentobarbital sodium (40 mg/kg), 26G and 24G needles were inserted into the left common carotid artery and the penile corpus cavernosum, respectively. A pressure transducer preconnected with heparin was used to monitor the mean arterial pressure (MAP) and maximum intracavernosal pressure (ICPmax) of the rats. The cavernous nerve of the penis was located behind the prostate and electrically stimulated (intensity, 3 V/5 V; frequency, 12 Hz; pulse width, 5 milliseconds; duration, 32 seconds; interval, 3 minutes). The ICPmax/MAP was recorded with a BL420 biological signal acquisition system (TECHMAN Technology Co, Ltd).

### Measurement of rat weight, serum testosterone concentration, and nitric oxide concentration in the penile corpus cavernosum

After abdominal anesthesia (1% sodium pentobarbital, 120 mg/kg), 2 mL of carotid artery blood was extracted and centrifuged. Rat serum testosterone was detected by enzyme-linked immunosorbent assay (ELISA) according to the instructions of the Rat Serum Testosterone Assay Kit (Lengton Bioscience Co, Ltd). The rat penile tissue was divided into 3 sections after removal of the foreskin, glans, and urethral spongiosum. These 3 sections were then examined by transmission electron microscopy, Western blot detection, and nitric oxide assays, respectively.^12^^,^^15^ Total protein was isolated from rat penile tissue by centrifugation and collected. Protein concentration was determined with a colorimetric method via a bicinchoninic acid protein detection kit (Beyotime Biotechnology Co, Ltd). The concentration of nitric oxide was measured with a colorimetric method (Elabscience Biotechnology Co, Ltd).^13^^,^^14^

### Serum levels of HS, HA, tumor necrosis factor α, and interleukin 6 in rats were measured by ELISA

Another 3 mL of carotid artery blood was taken from rats after euthanasia by overdosage of pentobarbital sodium, and the supernatant was aspirated after centrifugation. The serum levels of HS, HA, tumor necrosis factor α (TNF-α), and interleukin 6 (IL-6) were detected by ELISA according to the instructions of rat HS, HA, TNF-α, and IL-6 detection kits (Zhuo Cai Biotechnology Co, Ltd).

### Endothelial glycocalyx in the penile corpus cavernosum of rats was examined by electron microscopy

The midshaft cavernous tissue of rats was split into 0.2-cm tissue blocks and immersed in perfusion solution (2% glutaraldehyde, 2% sucrose, 0.1M disodium salt in methylamine hydrochloride buffer solution [pH 7.3], and 2% nitrate lanthanum) for 2 hours to fix the tissues. Penile tissue was soaked in solution without glutaraldehyde overnight and then placed in a solution containing 0.03 mol/L of NaOH and 2% sucrose solution for washing. After gradient dehydration with alcohol, the tissue was stained with uranyl acetate and lead citrate for sectioning (90 nm) and then observed under a transmission electron microscope for changes in the endothelial glycocalyx.^16^^,^^17^ All micrographs were taken at the same magnification (×12 000 and ×40 000). Five random locations throughout the entire cornea were selected where the basement membrane was sufficiently clear for vertical measurement. The thickness of each of the 5 regions was measured on each micrograph and averaged, and the range of thickness was determined.^18^^,^^19^

### Expression of endothelial nitric oxide synthase, phospho–endothelial nitric oxide synthase, syndecan 1, and heparanase in rat penile cavernous tissue detected by Western blot

The tissue of the rat penis corpus was crushed in a mortar cooled with liquid nitrogen. The crushed tissue was weighed, and lysis buffer at a ratio of 1 mg to 7.5 mL was added and incubated on ice for 1 hour, followed by ultrasound disruption for 3 minutes. The solution was centrifuged at low temperature, and the supernatant was aspirated. A bicinchoninic acid assay was conducted (Beyotime Biotechnology Co, Ltd) to determine the total protein concentration. Protein loading buffer (5×; Beyotime Biotechnology Co, Ltd) at a ratio of 4:1 was added and shaken well. The proteins were denatured at 98 °C for 15 minutes and stored at –20 °C for Western blotting detection. A 10% sodium lauryl sulfate–polyacrylamide gel was prepared according to the instructions, after which the proteins were separated via electrophoresis. After transfer, the membrane was removed, and 5% skim milk was added at room temperature. The membrane was incubated with shaking for 1 hour. Then, the polyvinylidene difluoride membrane was washed 3 times with Tris-buffered saline and Tween 20 for 10 minutes each time. Goat anti-rat (1:1000; Beyotime Biotechnology Co, Ltd) and goat anti-rabbit secondary (1:1000; Beyotime Biotechnology Co, Ltd) antibodies were added and incubated at room temperature for 1 hour, after which the membranes were incubated again after washing. An enhanced chemiluminescence reagent was used to visualize the bands. ImageJ version 1.8.0 (National Institutes of Health) was used for image acquisition and analysis.^12^

### Statistical analysis

Statistical analysis was performed with Prism version 9 (GraphPad Software LLC). The Shapiro-Wilk test was used to test the normality of the data. The data were analyzed with the Brown-Forsythe test or Bartlett’s test, and the results are expressed as mean ± SD. One-way analysis of variance was used to compare the groups. Tukey’s test was used as a post hoc test, and the difference was considered statistically significant at *P* < .05.

## Results

### Body weight and ICPmax/MAP

**Figure 1:**
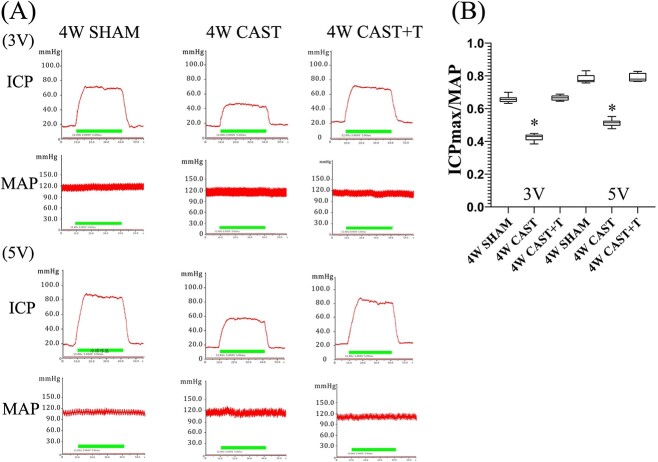
ICPmax/MAP for each group. (A) Traces of the ICPmax and MAP electrical stimulations at 3 V and 5 V among all groups. The ICPmax/MAP was significantly decreased in the castrated rats (mean ± SD; 0.42 ± 0.023, 3 V; 0.51 ± 0.024, 5 V) when compared with the castration + T group (0.66 ± 0.016, 3 V; 0.79 ± 0.027, 5 V) and the sham group (0.66 ± 0.020, 3 V; 0.78 ± 0.027, 5 V). (B) Data of ICPmax/MAP are represented by box and whisker plots. ^*^P < .01 vs sham and castration + T. ICP, intracavernous pressure; MAP, mean arterial pressure; T, testosterone.

The body weights of the castrated rats were not significantly different from those of the sham-operated rats or the testosterone replacement rats. There was no significant difference in the MAP among the groups of rats under electrical stimulation (3 V and 5 V; Table 1). The ICPmax/MAP ratio in the castration group was significantly lower than that in the sham-operated group and the castration + testosterone replacement group (*P* < .01; Figure 1). There was no significant difference in blood glucose levels among these groups (supplementary material).

**Table 1:** Rat penile corpus cavernosum results for each group.^a^

Group	**Body weight, g**	**T, nmol/L**	**NO, μmol/gprot**	**HS, ng/mL**	**HA, μg/mL**	**TNF-α, pg/mL**	**IL-6, pg/mL**
Sham	326.03 ± 6.73	20.29 ± 1.07	16.96 ± 0.47	7.400 ± 1.120	4.806 ± 0.440	20.22 ± 1.04	8.48 ± 0.67
Castration	319.29 ± 8.69	1.60 ± 0.15^*^	10.17 ± 1.24^*^	18.711 ± 1.353^*^	9.326 ± 0.562^*^	27.76 ± 1.09^*^	14.40 ± 0.80^*^
Castration + T	321.50 ± 9.81	19.33 ± 0.89	16.79 ± 0.80	11.706 ± 1.303^*^^#^	6.773 ± 0.545^*^^#^	22.97 ± 1.73^*^^#^	11.33 ± 0.99^*^^#^

Abbreviations: HA, hyaluronic acid; HS, heparan sulfate; IL-6, interleukin 6; NO, nitric oxide; T, testosterone; TNF-α, tumor necrosis factor α.

a Data are presented as mean ± SD (n = 6 rats per group).

^*^ *P* < .01 vs 4-week sham group.

# *P* < .05 vs 4-week castration group.

### Serum testosterone, nitric oxide, HS, HA, TNF-α, and IL-6 levels

**Figure 2:**
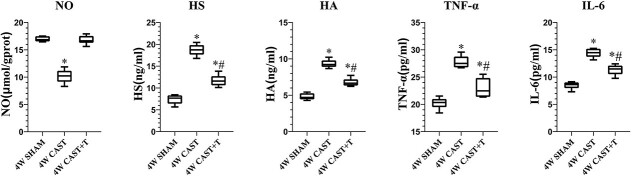
NO concentration in rat penile corpus cavernosum tissue and serum levels of HS, HA, TNF-α, and IL-6. ^*^P < .05 vs sham and castration + T. ^#^*P* < .01 vs castration. HA, hyaluronic acid; HS, heparan sulfate; IL-6, interleukin 6; NO, nitric oxide; TNF-α, tumor necrosis factor α.

The serum testosterone concentration in the castrated group (1.60 ± 0.15 nmol/L) was significantly lower than that in the sham-operated group (20.29 ± 1.07 nmol/L) and the castrated + testosterone replacement group (19.33 ± 0.89 nmol/L, *P* < .01). The nitric oxide concentration in the corpus cavernosum of the castrated group (10.17 ± 1.24 μmol/gprot) was significantly lower than that in the sham-operated group (16.96 ± 0.47 μmol/gprot) and the castrated + testosterone replacement group (16.79 ± 0.80 μmol/gprot, *P* < .01; Table 1). The serum levels of HS, HA, TNF-α, and IL-6 in castrated rats were significantly higher than those in the sham-operated group and the castration + testosterone replacement group (*P* < .01; Figure 2). The levels of HS, HA, TNF-α, and IL-6 in serum in the castration + testosterone replacement group were significantly increased when compared with the sham-operated group (*P* < .01).

### Electron microscopy observation of the endothelial glycocalyx in rat penile tissue

**Figure 3:**
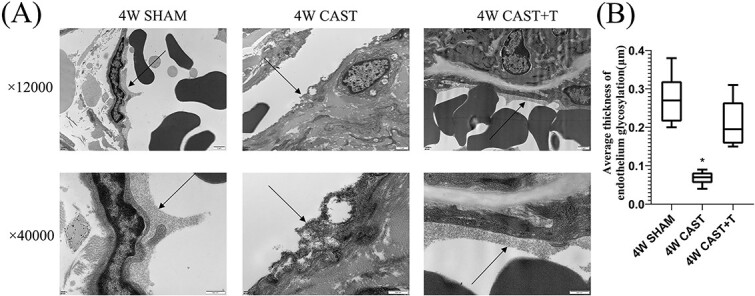
The endothelial glycocalyx in the corpus cavernosum of the penis of rats in each group observed under electron microscopy. (A) The vascular endothelial glycocalyx levels in the castration group (mean ± SD, 0.068 ± 0.017 μm) was significantly lower than that in the sham-operated group (0.273 ± 0.064 μm) and castration + testosterone replacement group (0.210 ± 0.062 μm). (B) The data represent the endothelial thickness of each group of blood vessels. Black arrows indicate the endothelial glycocalyx. ^*^P < .01 vs sham and castration + testosterone.

In the sham-operated group and the castration + testosterone replacement group, the endothelial glycocalyx was evenly and continuously distributed on the surface of the cavernous vascular lumen of the penis. However, after 4 weeks of castration, the endothelial glycocalyx on the surface of the cavernous vascular lumen of the penis was discontinuous and uneven in thickness. The thickness of the endothelial glycocalyx on the cavernous vessels of the penis in the castration group (0.068 ± 0.017 μm) was significantly lower than that in the sham-operated group (0.273 ± 0.064 μm) and the castration + testosterone replacement group (0.210 ± 0.062 μm, *P* < .01). There was no significant difference in endothelial glycocalyx thickness of penile cavernous tissue in the sham-operated group vs the castration + testosterone replacement group (Figure 3).

### Expression levels of endothelial nitric oxide synthase, phospho–endothelial nitric oxide synthase, syndecan 1, and heparanase in penile cavernous tissue

**Figure 4:**
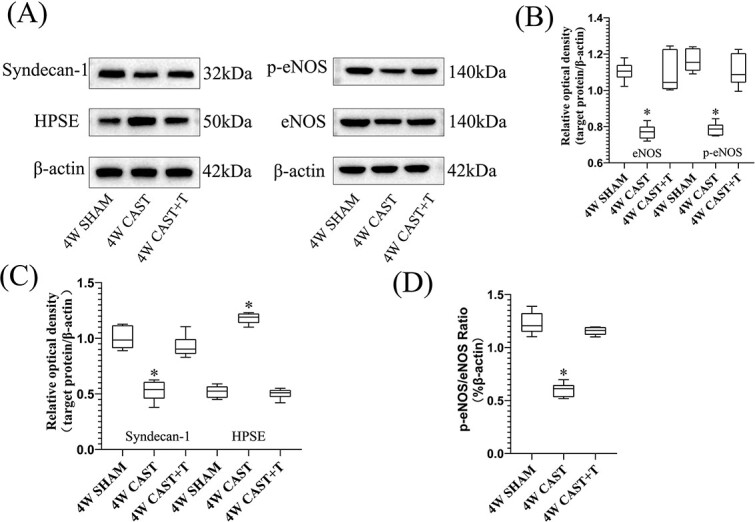
The expression of the target protein in the penile cavernous tissues of rats. (A) The expression of syndecan 1, eNOS, p-eNOS, and HPSE in the corpus cavernosum by Western blotting. (B) Box and whisker plots show the relative density values of eNOS and p-eNOS. The expression of eNOS and p-eNOS was greatly reduced in the castrated rats (mean ± SD; eNOS, 0.77 ± 0.04; p-eNOS, 0.79 ± 0.04) by comparison with those in the sham group (eNOS, 1.1.0 ± 0.05; p-eNOS, 1.16 ± 0.06) and cast + T group (eNOS, 1.09 ± 0.11; p-eNOS, 1.11 ± 0.09; *P* < .05). (C) The expression of syndecan 1 in the cast group was significantly lower than that in the sham-operated group and cast + T group (*P* < .05); the expression of HPSE in the cast group was significantly higher than that in the sham-operated group and cast + T group (*P* < .05). (D) p-eNOS/eNOS ratio (*P* < .05). cast, castration; eNOS, endothelial nitric oxide synthase; HPSE, heparanase; p-eNOS, phospho-eNOS; T, testosterone.

Levels of syndecan 1, phospho–endothelial nitric oxide synthase (p-eNOS), and endothelial nitric oxide synthase (eNOS) and the p-eNOS/eNOS ratio in the penis cavernous tissue of the castration group were significantly lower than those in the sham-operated group and the castration + testosterone replacement group (*P* < .05). Syndecan 1, p-eNOS, and eNOS levels and the p-eNOS/eNOS ratio in the penis cavernous tissue of the sham-operated group were not different from those of the castration + testosterone replacement group. The level of heparanase in the penis cavernous tissue of the castration group was significantly higher than that in the sham-operated group and the castration + testosterone replacement group (*P* < .05). The level of heparanase in the penis cavernous tissue of the castration group was not different from that in the castration + testosterone replacement group (Figure 4).

## Discussion

The present study showed that the serum testosterone concentration, nitric oxide levels, and ICPmax/MAP (3 V, 5 V) in the penile corpus cavernosum of the castration group were significantly lower than those in the sham-operated group and the testosterone replacement group (*P* < .05), indicating that low androgen status may reduce the nitric oxide level in the cavernous tissue of rats and impair erection function. Testosterone supplementation improved erectile function in castrated rats. This is consistent with previous research.^17^

In the study, the endothelial glycocalyx covering the medial aspect of the vascular lumen of the penile corpus cavernosum was observed for the first time by transmission electron microscopy. The endothelial glycocalyx is uniformly and continuously distributed on the surface of the cavernous vascular lumen of the penile corpus cavernosum under physiologic conditions, and the thickness is approximately 0.273 ± 0.064 μm. The thickness of the endothelial glycocalyx was significantly lower in the 4-week castration group (0.068 ± 0.017 μm) when compared with the sham-operated group. The breakdown of the endothelial glycocalyx leads to disruption of its continuity. The endothelial glycocalyx on the inner surface of the penile cavernous vessels recovered its continuous distribution after testosterone replacement therapy. The thickness of the endothelial glycocalyx was significantly higher in the 4-week castration + testosterone replacement group as compared with the castration group. This finding indicates that the endothelial glycocalyx on the inner surface of the penile cavernous tissue is disrupted or degraded in patients with a low androgen status. However, after androgen replacement therapy, the structure of the endothelial glycocalyx may be restored. Androgen may be an important factor in maintaining the endothelial glycocalyx of the penile cavernous tissue.

Syndecan 1 is an important core protein in the endothelial glycocalyx. Heparanase is an important protease that breaks the endothelial glycocalyx into HS and HA. Syndecan 1 forms vascular endothelial glycocalyx by binding to important branch chains, such as proteoglycans and glycosaminoglycans, and attaching to the surface of vascular endothelial cells.^20^ Vascular endothelial glycocalyx protects endothelial cells by reducing blood flow impingement on endothelial cells, while it activates endothelial cells by transducing vascular mechanical shear to upregulate the expression of eNOS and the level of nitric oxide.^21^^,^^22^ The expression of syndecan 1 and p-eNOS/eNOS was significantly lower in the castration group than the sham-operated group (*P* < .05). This suggests that the vascular endothelial glycocalyx became thinner after castration and that the mechanotransduction of the vessels decreased, leading to a decrease in the number of activated endothelial cells. It reduced the ratio of p-eNOS/eNOS and nitric oxide synthesis. The expression of heparanase in the penile tissue of rats in the castrated group was significantly higher than that in the sham-operated group. The levels of HS and HA in the serum of castrated rats were significantly higher than those in the sham-operated group. This suggests that the vascular endothelial glycocalyx was decomposed under low androgen status and that its structure was damaged. The expression of TNF-α and IL-6 was significantly higher in the castration group than in the sham-operated group. This suggests that the hypogonadal state causes an increase in inflammatory factors such as TNF-α and IL-6. The inflammatory factors activate the endothelial cells. This process leads to swelling of the endothelial cells and disruption of the connection between the endothelial cells and the endothelial glycocalyx, which accelerates the degradation of the endothelial glycocalyx.^23–25^ The p-eNOS/eNOS ratio in the penile vasculature of rats increased significantly after testosterone replacement therapy.^26^^,^^27^ Meanwhile, the levels of serum HS and HA, TNF-α, and IL-6 markedly decreased. This suggests that the endothelial glycocalyx on the surface of the cavernous vascular lumen of the penis requires androgen maintenance. Androgen deficiency may cause endothelial glycocalyx degeneration. It is suggested that the levels of IL-6, IL-1α, IL-1β, and TNF-α in serum in the castration + testosterone replacement group are significantly higher than those in the control group after 8 weeks of castration.^28^ The levels of IL-6 and TNF-α in the castration + testosterone replacement group were significantly reduced as compared with the castration group and significantly increased vs the control group after 4 weeks of castration in this study. The high levels of IL-6 and TNF-α can degrade the endothelial glycocalyx and lead to an increase of HA and HS.^29–31^ This indicates that low testosterone levels and an inflammatory response are important factors in the damage of the endothelial glycocalyx.

Low androgen concentration causes the breakdown of the endothelial glycocalyx in the endothelial cells of the cavernous blood vessels in the penis. Degradation of the endothelial glycocalyx can cause an acceleration in vascular permeability, edema, and inflammatory reactions, leading to loss of vascular responsiveness and low perfusion.^32^ Yet, apoptosis occurs in the endothelial cells of the penile corpus cavernosum after castration.^33^^,^^34^ These may be important causes of ED after castration. This series of signaling pathways requires further investigation.

In conclusion, a low androgen level can impair erection function by increasing the degradation of the endothelial glycocalyx in penile cavernous tissue. Androgen replacement therapy restores the structure of the endothelial glycocalyx in the penile corpus cavernosum of castrated rats and improves their erectile function.

The use of androgen receptor gene knockout methods may further clarify the relationship between androgen and the endothelial glycocalyx. Identifying drugs that upregulate endothelial glycocalyx gene expression may lead to the development of treatments for ED in patients who need to maintain low androgen levels.

## Supplementary Material

supplementary_materials_qfae039
