# Supplementary material for: A low androgenic state inhibits erectile function by suppressing endothelial glycosides in the penile cavernous tissue of rats
Source: Sex Med. 2024 Jun 15;12(3):qfae039. doi: 10.1093/sexmed/qfae039 (PMC11179729; doi:10.1093/sexmed/qfae039)
Supplement: supplementary_materials_qfae039 [file supplementary_materials_qfae039.docx]

Blood glucose in each group of rats ($\bar{x}\pm SD$)

| Blood glucose | | | | |
| --- | --- | --- | --- | --- |
| Group | 1W | 2W | 3W | 4W |
| SHAM | 5.71±0.75 | 5.59±0.45 | 5.67±0.12 | 5.75±0.57 |
| CAST | 5.55±0.93 | 5.56±0.48 | 5.36±0.33 | 5.58±0.65 |
| CAST+T | 5.88±0.60 | 5.79±0.37 | 5.77±0.30 | 6.00±1.05 |

Abbreviations: SHAM, sham operation; CAST, castration; CAST+T, castration + testosterone; 1W, 2W, 3W, 4W represents molding time.
